# Imbalanced Dermic Microbiome Aggravates Inflammation in Toenail Paronychia

**DOI:** 10.3389/fcimb.2021.781927

**Published:** 2021-12-03

**Authors:** Ying Li, Han Ma, Liang Xue, Huizhen Chen, Rui Pang, Yanyan Shang, Juan Luo, Xinqiang Xie, Jumei Zhang, Yu Ding, Moutong Chen, Juan Wang, Qingping Wu

**Affiliations:** ^1^ Guangdong Provincial Key Laboratory of Microbial Safety and Health, State Key Laboratory of Applied Microbiology Southern China, Guangdong Institute of Microbiology, Guangdong Academy of Sciences, Guangzhou, China; ^2^ Department of Dermatology, The Fifth Affiliated Hospital of Sun Yat-Sen University, Zhuhai, China; ^3^ School of Biology and Biological Engineering, South China University of Technology, Guangzhou, China

**Keywords:** dermis, microbiome, paronychia, inflammation, anaerobic microbes

## Abstract

The commensal microbiome influences skin immunity, but its function in toenail health remains unclear. Paronychia is one of the most common inflammatory toenail diseases, but antibiotic treatment is seldom effective in clinical cases. In this study, we performed *16S rRNA* sequencing to investigate the characteristics of microbes associated with paronychia in order to identify the key microorganisms involved in inflammation. Seventy dermic samples were collected from patients with paronychia and the differences in dermic microbiota were analyzed in patients with different inflammation severities. Distinct clustering of dermal microbiota was observed in the dermis with different inflammation severities. A higher relative abundance of anaerobic microorganisms such as *Parvimona*, *Prevotella*, and *Peptoniphilus* was observed in severe paronychia, whereas *Lactobacillus* disappeared with disease progression. Co-occurring network analysis suggested that the disturbance of the dermic microbiome and attenuation of antagonism by *Lactobacillus* against anaerobic pathogens may aggravate inflammation in paronychia. Functional analysis showed that dermic microbiome disturbance may worsen microbial metabolism and tissue repair in the skin. In conclusion, we revealed that an increased abundance of anaerobic microorganisms and loss of *Lactobacillus* in the dermis may promote paronychia progression and microbiological imbalance may aggravate inflammation in patients with paronychia.

## 1 Introduction

Paronychia is one of the most common inflammatory diseases of the periungual folds, which arises as a result of ingrown nails (onychocryptosis), neglected wounds, dermatological conditions such as Reiter’s syndrome, and medications such as retinoids (Rockwell, 2001; Duhard, 2014; Relhan et al., 2014; Lomax et al., 2016). Inadequate treatment can result in the transformation of the infection from a local disease (flexor tenosynovitis, osteitis, or osteoarthritis) to a systemic disease (endocarditis or bacterial translocation) (Pierrart et al., 2016). *Staphylococcus aureus* and *Streptococcus pyogenes* are regarded as the causative pathogens, and bacterial infections are key factors of inflammatory aggravation in paronychia (Marten, 1959; Geizhals and Lipner, 2019). Despite the strong correlation between infective microorganisms and inflammation in paronychia, antibiotic treatment alone cannot control the disease, and it may lead to disease recurrence in clinical practice (Laxton, 1995; Park and Singh, 2012). Several studies have shown that surgery, drainage, and laser treatment may relieve the symptoms and reduce the recurrence rate of paronychia without antibiotic medication, but the underlying mechanisms of these treatments remain unknown (El-Komy and Samir, 2015; Ferreira Vieira d'Almeida et al., 2016; Pierrart et al., 2016).

The skin microbiome is an ecosystem comprising hundreds of diverse bacteria that interact with the host epithelial and immune cells (Fredricks, 2001; Chaudhari et al., 2020; Flowers and Grice, 2020). Commensal microbiota on the skin are involved in both innate and adaptive immunity and maintain the integrity of the skin barrier against pathogens (Cogen et al., 2008; Dréno et al., 2016; Zhai et al., 2018; Chaudhari et al., 2020; Flowers and Grice, 2020). For instance, *Lactobacillus acidophilus* secretes antibacterial agents on the skin and prevents the adhesion of pathogens, whereas *Staphylococcal* species can inhibit the Toll-like receptor-dependent pathway and modulate inflammation on the skin (Coconnier et al., 2000; Lai et al., 2009; Atabati et al., 2020; Williams et al., 2020). Major technical and analytical breakthroughs have enabled scientists to investigate the microbiome at the genetic level, and studies have demonstrated that the influence of nonculturable microorganisms on the skin ecosystem is often underestimated (Chen et al., 2018; Flowers and Grice, 2020). Recently, *Fusobacterium* has been suggested to promote melanoma development and induce atopic dermatitis development, and *Porphyromonas* and *Peptoniphilus* might lead to skin lesions in hidradenitis suppurativa, indicating that the modulation of the microbiome can serve as a strategy to treat skin diseases (Guet-Revillet et al., 2017; Ring et al., 2017; Mrázek et al., 2019; Langan et al., 2020).

The unique composition of the microbiome has been determined in the toenails of healthy volunteers, revealing a high abundance of *Staphylococcus* and *Corynebacterium*; however, the relationship between the microbiome and toenail health remains unclear (Grice et al., 2009; Wałaszek et al., 2018). In this study, we investigated the changes in the dermic microbial communities in patients with different severities of paronychia, to identify key microbiological factors that are related to inflammation in toenail disease.

## 2 Materials and Methods

### 2.1 Study Participants and Sample Collection

#### 2.1.1 Ethical Compliance

Seventy acute paronychia patients with onychocryptosis were recruited from the Department of Dermatology, the Fifth Affiliated Hospital of Sun Yat-sen University from March 2019 to March 2020. The inclusion criteria for the participants were as follows: (i) over 18 years; (ii) the necessity of surgical treatment assessed by two experienced dermatologists; (iii) no pregnancy; (iv) no history of malignant diseases, autoimmune diseases, or diabetes; and (v) no exposure to antibiotics (systemic or topical) within the past 2 months. This study was approved by the Ethics Committee of the Fifth Affiliated Hospital of Sun Yat-sen University (project K148-1).

All participants underwent a comprehensive examination and the severity of paronychia was evaluated using the nail psoriasis severity index (NAPSI) (Rich and Scher, 2003; Atış et al., 2018). In accordance with the NAPSI index, patients with paronychia were assigned to mild inflammation group (NAPSI score ≤ 2) and severe inflammation group (NAPSI score = 3). Among the 70 patients, 35 were assigned to the severe group and 35 to the mild group.

#### 2.1.2 Surgical Procedures and Sample Collection

All patients with paronychia received surgical treatment according to the method described previously (Ma, 2021). Briefly, an arc incision was made along the lateral nail fold to remove the hypertrophic tissue and corresponding nail matrix, and the proximal lateral nail fold was sutured subsequently.

The dermic tissues along the nail fold were separated and washed gently with 2 mL of phosphate buffered saline (PBS) to remove the blood and nail tissues. The clean tissues were stored in Sample Protector of RNA/DNA (Takara Bio, Shiga, Japan), immediately transported to the laboratory, and stored at -80°C until nucleic acid extraction.

### 2.2 Microbial DNA Extraction and 16S rRNA Amplification

The tissues were gently washed twice with 1 mL of PBS to remove the Sample Protector of RNA/DNA. Microbial DNA was extracted from the dermic tissues using the QIAamp PowerFecal DNA kit (Qiagen, Hilden, Germany) according to the manufacturer’s instructions. The concentration and quality of the microbial DNA were determined using Nanodrop (Thermo Scientific, Wilmington, USA) and by 0.6% agarose gel electrophoresis, respectively. The microbial DNA with high integrity (average fragment size > 30 kb) and purity (OD 260/280 of 1.8 and OD 260/230 of 2.0–2.2) was diluted to 20 ng/μL for amplification.

The V3-V4 hypervariable regions of the 16S rRNA gene were amplified using the primers 341F 5′-CCTACGGGNGGCWGCAG-3′ and 806R 5′-GGACTACHVGGGTWTCTAAT-3′ (Fadrosh et al., 2014). Polymerase chain reaction (PCR) was performed using the UCP Multiplex PCR Kit (Qiagen). Samples were denatured at 95°C for 3 min, followed by 16 cycles at 95°C for 30 s, 55°C for 30 s, and 72°C for 30 s, with a final elongation at 72°C for 5 min. The PCR products were purified using AMPure XP DNA clean beads (Beckman, Greater Los Angeles, CA, USA). The amplicon libraries were generated using the QIAseq Ultralow Input Library Kit (Qiagen).

### 2.3 Illumina Miseq Sequencing

An Agilent Bioanalyzer 2100 system (Agilent Technologies, Santa Clara, CA, USA) and Qubit™ dsDNA HS Assay Kit (Thermo Fisher Scientific, Waltham, MA, USA) were used to validate the amplicon library. Paired-end sequencing was conducted on the Miseq platform with the Miseq Reagent Kit version V3 (2 × 300bp Paired-End Reads, Illumina, San Diego, CA, USA).

### 2.4 Processing of Sequencing Data

The raw data were transformed into fastq files according to their index sequences by Miseq Reporter (Illumina). Primary fastq files were trimmed and quality-filtered using CLC genomic workbench 20.0 with the Microbial Genomics Module (Qiagen). The trimmed sequences were matched to those in the Greengenes 13.5 database with 97% similarity cutoff.

### 2.5 Bioinformatic and Statistical Analyses

Taxonomy analysis of the amplicon sequences and statistical analysis were performed using Calypso software version 8.84 (Zakrzewski et al., 2017). Alpha diversity was determined based on the Shannon index and phylogenetic diversity, which were assessed using the analysis of variance. Microbial diversity was also investigated using redundancy analysis (RDA) based on the disease severity group. The core microbiome was identified as described by Ainsworth et al. (2015).

Key taxonomic discovery was performed using linear discriminant analysis effect size (LEfSe) (Segata et al., 2011). In brief, non-parametric factorial Kruskal–Wallis sum-rank test was first applied to identify significant differential abundance at different taxonomy levels between different inflammatory groups, and then linear discriminant analysis (LDA) was used to estimate the effect size of each differentially abundant feature. In our study, the α value of 0.05 was set for the factorial Kruskal–Wallis test and the threshold on the logarithmic LDA score of 2.0 was set for discriminative features. The results of the LEfSe analysis were visualized by cladograms, in which the taxonomy relationship of all tested dermic microorganisms was shown from kingdom, phylum to genus and species. Severe paronychia-specific microbes were marked red in the cladograms, whereas mild paronychia-specific microbes were marked green.

A network analysis was performed to identify co-occurring and exclusive relationships among the core microorganisms in the dermis. Taxa were represented as nodes, taxa abundance as node size, and edges depicted positive (green) and negative (red) associations. Networks were generated by computing the associations between taxa by Pearson’s correlation and nodes were colored based on their association with the severity of paronychia. Only significant relationships (*P* < 0.05) were visualized in the network. The network was visualized using Cytoscape v3.9.0 (Assenov et al., 2008).

Metagenome prediction was performed using the amplicon sequencing approach in the phylogenetic investigation of communities by reconstructing unobserved states (PICRUSt) (Langille et al., 2013). Significant differences between different severity groups were examined at the different Kyoto Encyclopedia of Genes and Genomes (KEGG) pathway hierarchy level using the analysis of variance.

A correlation analysis was carried out between the key genera and metabolic pathways in the dermic samples of patients with mild paronychia using Pearson’s correlation test.

In all statistical analysis, *P <* 0.05 was considered to indicate significance, and *P* values were adjusted using the false discovery rate, Bonferroni correction, or area under the curve correction in different statistical models.

## 3 Results

### 3.1 Clinical and Pathological Features of Patients With Paronychia

The dermic microbiome was characterized in 35 paronychia patients with a mild inflammation (32 males and 3 females, aged 18–42 years) and in 35 patients with a severe inflammation (30 males and 5 females, aged 18–38 years). No significant difference in sex, age, and living habits (smoking and drinking) was found between the groups. Detailed information of the patients is shown in **Table 1**.

**Table 1:** Clinical characteristics of patients with paronychia.

	Mild (n = 35)	Severe (n = 35)	*P*
**Median Age** (years) (range)	46 (18–51)	50 (18–69)	0.430
**Sex**			
Male (cases) (%)	18 (51.42)	27 (80.00)	0.626
Female (cases) (%)	17 (48.57)	7 (20.00)	
**History of smoking** (case) (%)	4 (11.43)	12 (3.75)	0.403
**Paronychia severity index**			
Nail fold	2.83 ± 0.02	2.85 ± 0.32	0.097
Edema	2.45 ± 0.34	2.95 ± 0.12	0.047
Erythema	1.20 ± 0.03	2.92 ± 0.14	<0.001
Nail plate changes	2.64 ± 0.24	3.02 ± 026	0.027
Cuticle	0.36 ± 0.41	2.31 ± 0.56	<0.01

Pathological examination was performed to confirm the disease severity in each patient. In the skin tissue of patients in the severe group, diffuse infiltration of lymphocytes, histocytes, and plasma cells was observed in the dermis, whereas only perivascular infiltration of a few neutrophils, lymphocytes, and histocytes was detected in the mild group patients.

### 3.2 Dermic Microbial Community Structure in Patients With Different Severities of Paronychia

**Figure 1:**
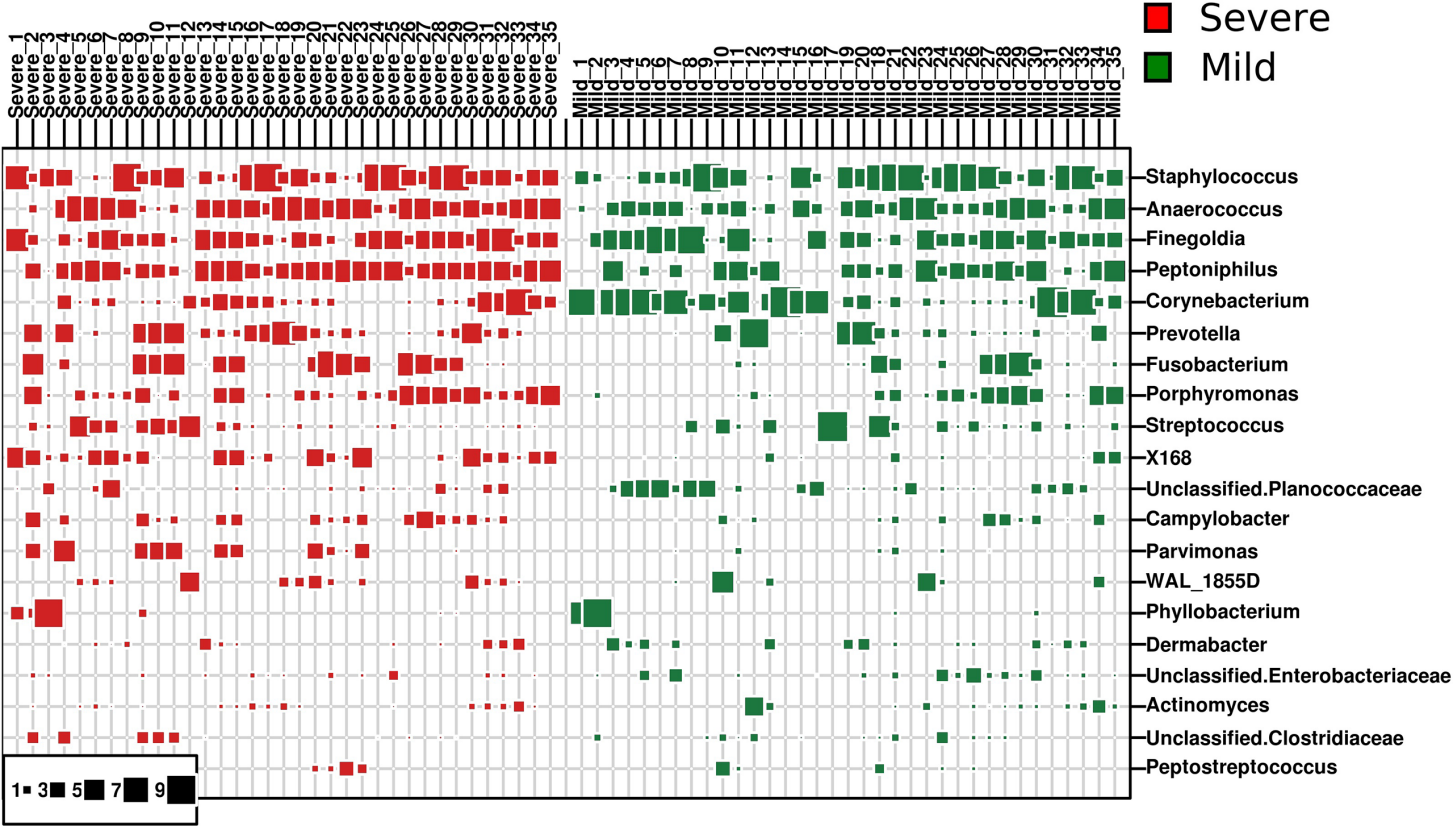
Dominant bacterial genera in the dermis of patients with paronychia. The top 20 dominant bacterial genera in dermic tissue are shown in the bubble chart, patients with mild paronychia are in the green group, and patients with severe paronychia are in the red group. The genera were listed from the top to the bottom according to their relative abundance in the dermic tissue, and their RAs are shown in squares.

Seven hundred and nineteen unique operational taxonomic units (OTUs) were identified among all samples, demonstrating the diversity of the bacterial community in paronychia tissue. *Staphylococcus* was the dominant genus in the dermis of the toenails, and its relative abundance (RA) was 17.21% ± 3.88% in the severe group and 19.50% ± 4.02% in the mild group (*P* = 0.683) (**Figure 1**). On the contrary, some microorganisms such as *Lactobacillus* (0.173% ± 0.030% in 15 mild patients) and *Brachybacterium* (0.032% ± 0.013% in 5 mild patients) could only be found in some dermic samples of patients with mild paronychia. The RAs of other dominant microorganisms are shown in **Table 2**. This result revealed that the composition of dermic microbiota changed during disease progression.

**Table 2:** Comparison of relative abundance of the 10 dominant dermic microbes in mild and severe paronychia.

Dominant Genus	Relative Abundance		*P*
	Mild (n = 35)	Severe (n = 35)	
***Staphylococcus***	19.50% ± 4.02%	17.21% ± 3.88%	0.683
***Anaerococcus***	8.42% ± 1.72%	16.18% ± 2.48%	0.012
***Finegoldia***	11.98% ± 2.74%	9.52% ± 1.70%	0.448
***Peptoniphilus***	7.06% ± 1.56%	13.32% ± 1.60%	0.007
***Corynebacterium***	19.68% ± 4.69%	4.26% ± 1.81%	0.003
***Prevotella***	0.01% ± 0.01%	0.00% ± 0.00%	0.725
***Fusobacterium***	2.98% ± 1.43%	8.21% ± 2.28%	0.056
***Porphyromonas***	2.70% ± 0.92%	4.52% ± 1.04%	0.194
***Streptococcus***	4.04% ± 2.62%	2.41% ± 1.03%	0.564
***X168***	0.30% ± 0.16%	3.89% ± 1.04%	0.001

**Figure 2:**
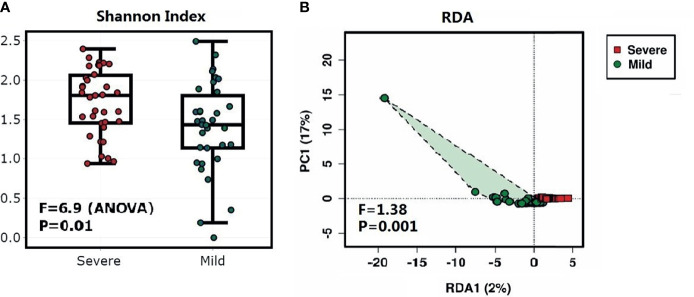
Diversity analysis of the dermic microbiome of patients with paronychia. ***(*A*)*** Comparison of alpha diversity of dermic microbiome using the Shannon’s α-index between patients with mild paronychia (green) and those with severe paronychia (red). **(B)** Redundancy analysis of dermic microbiome between the mild (green) and severe (red) paronychia groups.

The bacterial diversity was different in the dermis of patients with different severities of paronychia. The alpha diversity analysis revealed higher genus diversity in the dermis of patients with severe paronychia (*P* = 0.01), indicating more microorganisms were involved in inflammation progression. In addition, the redundancy analysis at the genus level revealed distinct clustering of the microbiota in different severity groups (*P* = 0.001) (**Figure 2**).

### 3.3 Key Microbiota Involved in the Aggravation of Inflammation in Paronychia

All patients with paronychia shared 10 genera in the core microbiota, namely *Anaerococcus*, *Corynebacterium*, *Finegoldia*, *Fusobacterium, Peptoniphilus*, *Porphyromonas*, *Prevotella*, *Staphylococcus*, *Streptococcus*, and an unclassified genus of Planococcaceae. Five genera of aerobic bacteria, namely *Dermabacter*, *Ochrobactrum*, *Pseudomonas*, an unclassified genus of *Aeromonadaceae*, an unclassified genus of *Enterobacteriaceae*, and 1 genus of the anaerobic bacteria *Lactobacillus* were identified as the unique core microbiota in patients with mild paronychia. On the contrary, four anaerobic or facultative anaerobic bacteria were identified as unique core microbiota in the severe group, namely, *Actinomyces*, *Campylobacter*, *Helcococcus*, and *X168*.

**Figure 3:**
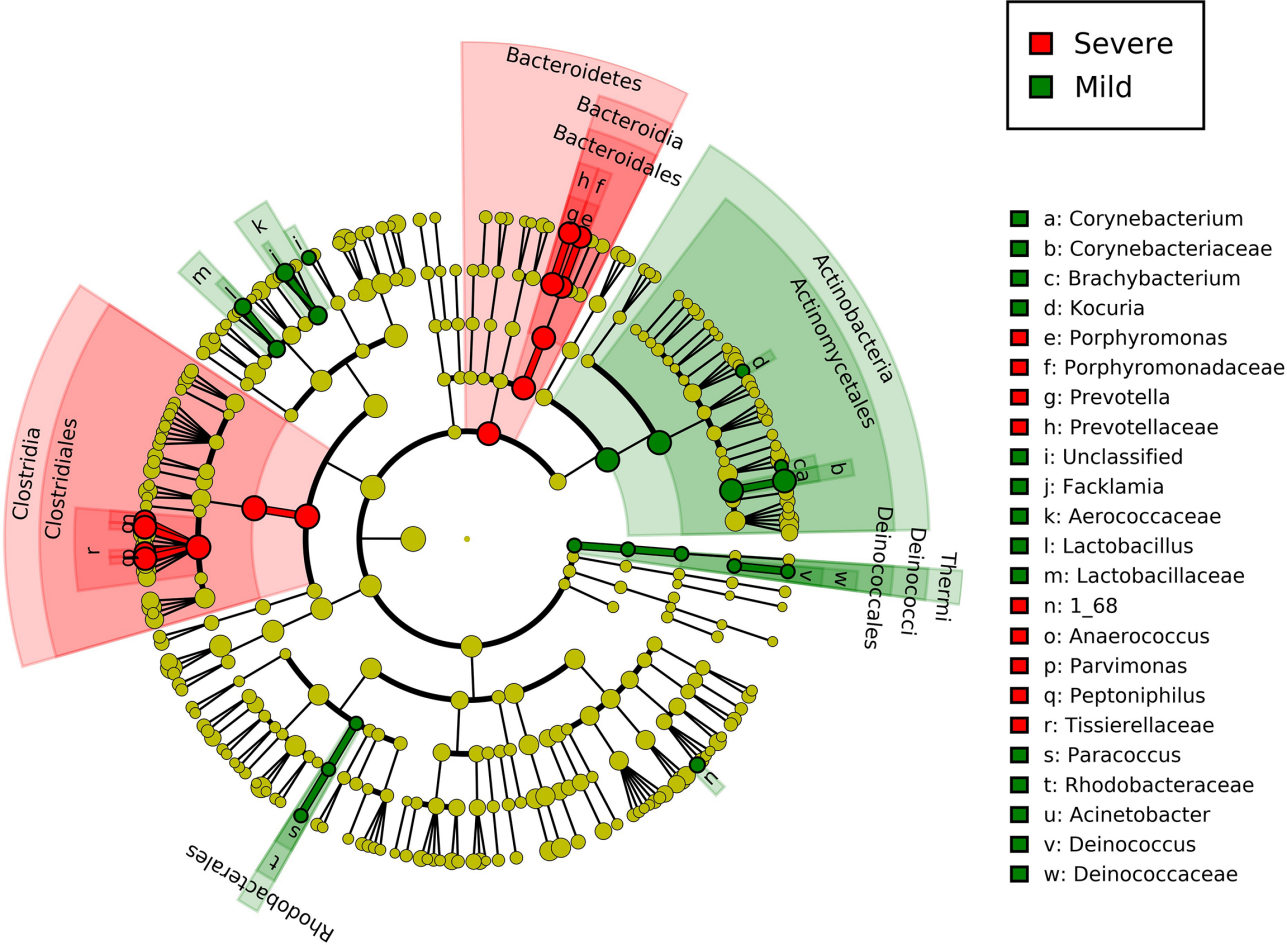
Linear discriminant analysis effect size of group-specific microbes in patients with different severities of paronychia. The inflammation-specific microbes with biomarker significance are shown in the colored taxonomy cladogram using the LEfSe analysis. The RA differences with P < 0.05 and LDA ≥ 2.0 were regarded as microbial features with discriminative significance. The severe paronychia-specific microbes are marked red in the cladograms and the mild paronychia-specific microbes are marked green.

The LEfSe biomarker discovery analysis revealed the specific microbiota at both genus and species levels (**Figure 3**). *Peptoniphilus*, *X168*, *Prevotella*, *Parvimonas*, and *Porphyromonas* were identified as biomarker microorganisms in the severe group of patients, whereas *Corynebacterium*, *Facklamia*, *Lactobacillus*, *Paracoccus*, *Kocuria*, *Bradyrhizobium*, *Brachybacterium*, *Acinetobacter*, and *Deinococcus* were identified as biomarker microorganisms in the mild group.

As *Staphylococcus* and *Streptococcus* are considered the most important pathogens in paronychia, the RA of these two genera were compared in the mild and severe paronychia groups, but no difference in RA was found between the groups (*Staphylococcus* 19.50% ± 4.02% in the mild group vs. 17.21% ± 3.88% in the severe group, P = 0.865 and *Streptococcus* 4.04% ± 1.50% in the mild group vs. 2.41% ± 0.61% in the severe group, *P* = 0.294, respectively). At the species level, *Staphylococcus aureus* was identified in both groups with no difference in RA (14.38% ± 3.66% in the mild group vs. 16.50% ± 3.88% in the severe group, *P* = 0.691), but the RA of an unclassified species of *Staphylococcus* was lower in the severe group (9.13% ± 3.44% in the mild group vs. 1.82% ± 0.62% in the severe group, *P* = 0.040). On the contrary, no sequence of *Streptococcus pyogene* was found in our research.

### 3.4 Dermic Microbiome Network in Paronychia and Its Functional Prediction in Inflammation

**Figure 4:**
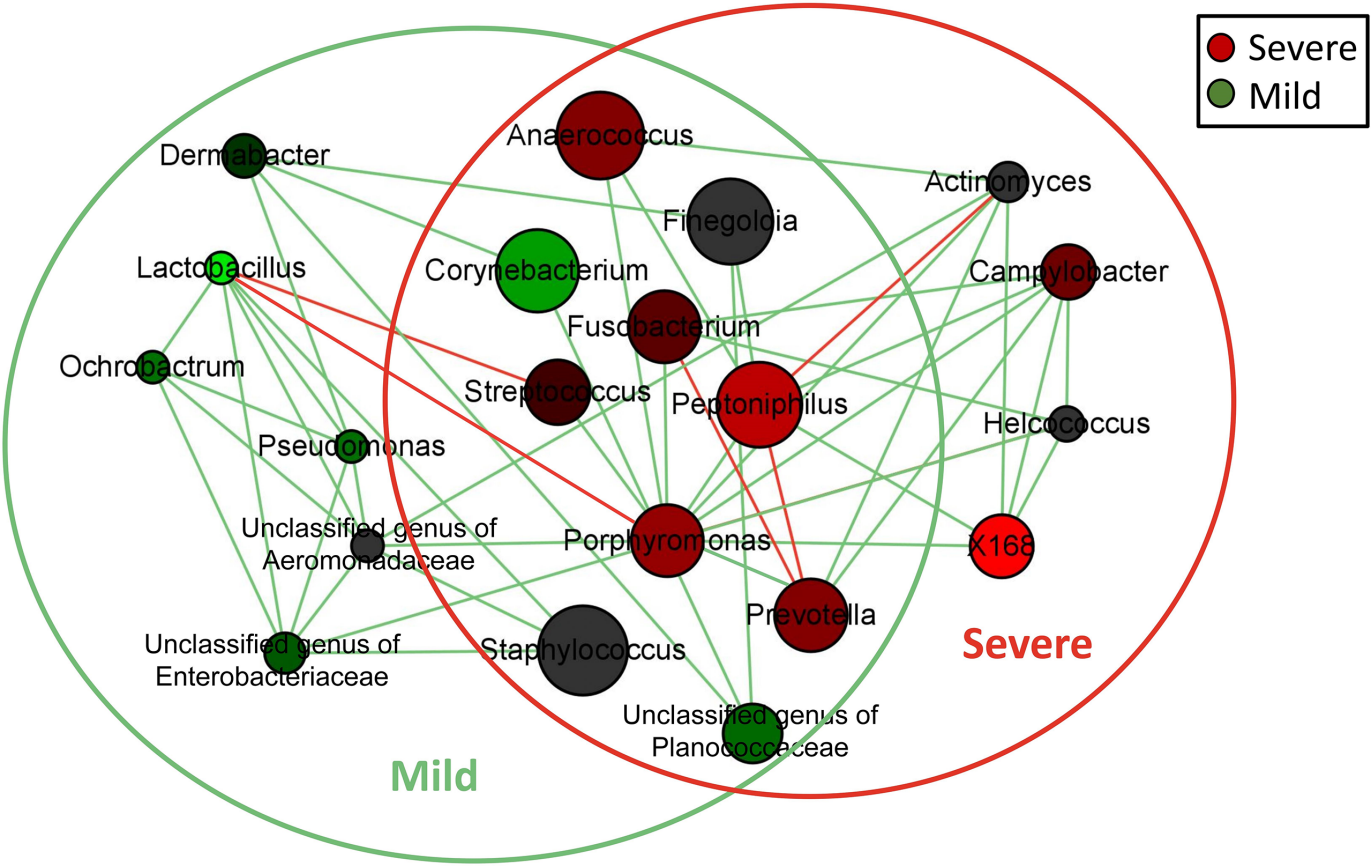
Network analysis of microbiome in patients with dermis paronychia. Co-occurrence relationship of the key dermic microorganisms in patients with different disease severities are shown at the genus level. Genus is presented as nodes, genus abundance is presented as node size, and edges are represented based on their association tested using Pearson’s correlation (positive inter-node correlations are green and negative inter-node correlations are red). Nodes in the green circle were the core dermic microbes in patients with mild paronychia, whereas nodes in the red circle were the core dermic microbes in patients with severe paronychia.

Network analysis was performed to compare the relationship of the dermic microbiome with mild and severe paronychia using tissues from patients with mild and severe disease. The co-occurrence relationship changed as inflammation aggravated in the dermis tissue (**Figure 4**). In the dermic microbiome of severe paronychia, positive associations with pathogenic anaerobes such as *Porphyromonas*, *Actinomyces*, *Prevotella*, and *X168* were observed. *Lactobacillus* was detected only in patients with mild paronychia and was negatively correlated with anaerobic pathogens such as *Porphyromonas* and *Streptococcus*.

**Figure 5:**
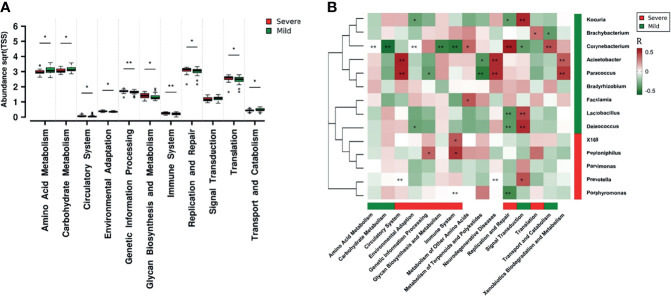
Dermic microbial function comparison of paronychia in patients with different inflammation severities and their relationship with the core microbiome. **(A)** Functional analysis was performed at the KEGG second hierarchy level in the dermic microbiota. Wilcoxon test was applied to compare each category of microbial function, and results with *P* < 0.05 were considered statistically significant. **(B)** Heatmap of correlation between the core microbiome and key metabolic pathway in the dermis of patients with mild paronychia. Core genera of dermic microbiome and their correlations with the 15 discrepant metabolite pathways in different groups were analyzed using Pearson correlation analysis. The Pearson correlation coefficient between the genus and the metabolic pathway was calculated; it is shown in a colored matrix. Red square represents a positive correlation, whereas green square represents a negative correlation. Red bar means the marked functions and genera were higher in the severe patients whereas green bar means the marked functions and genera were higher in the mild patients, with white bar indicating no significant difference between the groups.*P < 0.05, **P < 0.01.

Functional differences in the dermal microbiota between the mild and severe paronychia groups were analyzed at the KEGG second hierarchy level. The microbiologic functions with inter-group difference are shown in **Figure 5A**. In paronychia patients with a mild inflammation, dermic microorganisms showed better abilities in amino acid metabolism, carbohydrate metabolism, signal transduction, and metabolites transportation and catabolism. On the contrary, the dermal microbiota in the severe paronychia group showed higher parameters related to environmental adaptation, genetic information processing, glycan biosynthesis and metabolism, replication and repair, and translation, indicating the microorganisms had stronger abilities to survive in the tissue.

The correlation analysis in the dermis of patients with mild paronychia showed that most of these microbial functions were attributed to *Acinetobacter*, *Brachybacterium*, *Corynebacterium*, *Deinococcus*, *Kocuria*, *Lactobacillus*, *Paracoccus*, *Prevotella*, and *Porphyromonas*. As some microorganisms such as *Lactobacillus* and *Brachybacterium* disappeared in the dermis of patients with severe paronychia, the microbial functions including translation, replication, and repair might downregulate (**Figure 5B**). This result indicated that treating certain kinds of microorganisms may help to reconstruct the healthy microbial function in dermis.

## 4 Discussion

Paronychia refers to inflammation of the periungual folds that was previously recognized as being caused by aerobic microorganisms such as *S. aureus* and *S. pyogene* (Duhard, 2014; Lomax et al., 2016). However, in the early 1980s, some case reports have indicated anaerobic bacteria as the key pathogens in the progression of inflammation in paronychia (Whitehead et al., 1981; Brook, 1993). In this study, we evaluated the dermic microbiome of toenail paronychia tissue by *16S rRNA* gene sequencing and confirmed the diversity and complexity of the toenail ecosystem. In contrast to the microbial composition of healthy toenails (Grice et al., 2009; Dréno et al., 2016), the predominant microorganisms in paronychia were *Staphylococcus* and *Anaerococcus*, indicating that both aerobic and anaerobic bacteria cause inflammation in the toenail tissue. Our findings lend some support to the earlier case reports that anaerobic organisms such as *Anaerobic cocci*, *Bacteroides* species, and *Fusobacterium* species can also cause severe paronychia in the fingernails and toenails (Brook, 1990; Brook, 1993; Riesbeck, 2003; Brook, 2007). The unique and complex composition of microorganisms in the dermic tissue of paronychia described in our study explain the poor outcomes obtained when using narrow-spectrum antibiotics to alleviate inflammation caused by microorganisms, and treatment of paronychia should focus on the modulation of the microbiome structure in the toenail besides the elimination of pathogens.

We also found that the composition of the dermic microbiota changed with the aggravation of inflammation. The core dermic microorganisms shifted from aerobic to anaerobic as the inflammation progressed. Our results also revealed that anaerobic pathogens such as *Peptoniphilus* and *Porphyromonas* played an important role in paronychia tissues with a severe inflammation. Such changes in the dermic microbiome were similar to the etiology of hidradenitis suppurativa, in which *Peptoniphilus* and *Porphyromonas* species were related to skin lesions and abscesses (Brook, 2007; Ring et al., 2017; Langan et al., 2020). Our findings indicate that besides the widely recognized aerobic pathogens such as *S. aureus*, anaerobic microbes play an important role in inflammation progression in paronychia. Moreover, there was no difference in the abundance of *Staphylococcus* in the dermis of patients with mild and severe paronychia. As studies have proved that coagulase-positive and -negative *Staphylococcus* might have entirely different influences on the skin health (Nakatsuji et al., 2017), we believe a more precise detection such as antigen-capture PCR is needed to better identify the precise proinflammatory factor in paronychia.

Another microbial factor related to inflammatory aggregation in paronychia may be the loss of probiotics in the dermic tissue. *Lactobacillus* spp. and *Staphylococcus* spp. are important components of the skin microbiome (Grice et al., 2009; Mrázek et al., 2019), and some studies have confirmed their positive roles in regulating skin immune balance by generating active metabolites (Mu et al., 2018; Atabati et al., 2020). Studies have reported that *Lactobacillus* and *Staphylococcus* modulate skin immunity by producing active metabolites such as lipoteichoic acid and prevent the adhesion and colonization of pathogens (Cogen et al., 2008; Bodera and Chcialowski, 2009; Lai et al., 2009; Atabati et al., 2020). In our study, reconstruction of the microbial ecosystem in paronychia demonstrated the anaerobic pathogen antagonist potential of both *Lactobacillus* and *Prevotella*, and the loss of these microorganisms may attenuate the growth inhibition function of proinflammatory microbes such as *Streptococcus*, which aggravate inflammation in the toenail tissue. This finding indicates the importance of ecosystem reconstruction in paronychia and suggests the potential of probiotic supplementation for paronychia treatment.

Functional prediction of the dermic microbiome may improve our understanding of the inflammatory signals transduced by the pathogens to the toenail tissue. As anaerobic microbes accumulate in paronychia, the functional microorganisms involved in carbohydrate and amino acid metabolism as well as metabolite transport and catabolism reduce, leading to a decrease in metabolism in the skin ecosystem. When waste metabolites accumulate in the dermic tissue, they may serve as strong proinflammatory factors in innate immunity and induce cytokine storms in the toenail tissue (Belkaid and Tamoutounour, 2016; Byrd et al., 2018). Our study also suggested that the changes in microbiologic function in dermis were due to the changes in certain genera of pathogens such as *Brachybacterium*, *Corynebacterium*, *Acinetobacter*, *Paracoccus*, and *Facklamia*. The functional analysis of dermic microbiota offers a new insight into the pathogenesis of inflammation in paronychia and indicates the potential use of probiotics to improve metabolism in the skin system.

New genomic technologies may more comprehensively and precisely elucidate the microbiome changes in the dermis of patients with paronychia. In this study, we identified the potential proinflammatory microbes in paronychia and emphasized the importance of integrity of the microbiome in toenail health. Our results suggest that the imbalance of the anaerobic microbe in the dermic microbial community may hinder amino acid and carbohydrate metabolism in the skin and lead to the accumulations of waste metabolites, which serve as proinflammatory factors in the toenail tissue. However, the limited number of participants may have introduced a bias in statistical analysis and might have limited the generalizability to a larger population with paronychia. Furthermore, the sequencing of the V3-V4 region is not sufficient to identify the specific pathogenic species in paronychia. The proinflammatory mechanism of microorganisms was based on the model predicted by PICRUSt, and the results of experiments such as *Lactobacillus* supplementation to alleviate inflammation need to be verified.

## 5 Conclusions

In this study, we observed the relationship between dermic microbiome and inflammation in paronychia and explained the influence of microorganisms on the immune system in the dermis. The analysis of proinflammatory pathogens in paronychia revealed that in addition to well-known aerobic bacteria such as *Staphylococcus*, anaerobic microbes are also involved in disease progression. Our study demonstrates the importance of microbiome integrity in skin health and potential of probiotic supplementation for paronychia treatment; further research is needed to verify the anti-inflammatory effect of *Lactobacillus* in paronychia.

## Data Availability Statement

The datasets presented in this study can be found in online repositories. The names of the repository/repositories and accession number(s) can be found below: https://www.ncbi.nlm.nih.gov/genbank/, PRJNA765531.

## Ethics Statement

The studies involving human participants were reviewed and approved by the Ethics Committee of the Fifth Affiliated Hospital of Sun Yat-sen University. The patients/participants provided their written informed consent to participate in this study.

## Author Contributions

YL, HM, and QW designed the study. HM and JL recruited the clinical cohort, collected samples, and performed the follow-up surveys. YL, HC, and YS contributed to amplicon sequencing. YL, HC, LX, and RP analyzed the data. YL and XX wrote the manuscript. JZ, YD, MC, JW, and QW performed critical revisions of the manuscript. All authors contributed to the article and approved the submitted version.

## Funding

This work was supported by Guangdong Provincial Key Laboratory (2020B121201009), Research and Development Projects in Key Areas of Guangdong Province, China (2018B020205002) and Guangdong Provincial Academy of Sciences to Implement the Innovation Driven Development Capacity of Special Funds Project (2020GDASYL-20200301002). The funders had no role in the design of the study; in the collection, analyses, or interpretation of data; in the writing of the manuscript, or in the decision to publish the results.

## Conflict of Interest

The authors declare that the research was conducted in the absence of any commercial or financial relationships that could be construed as a potential conflict of interest.

## Publisher’s Note

All claims expressed in this article are solely those of the authors and do not necessarily represent those of their affiliated organizations, or those of the publisher, the editors and the reviewers. Any product that may be evaluated in this article, or claim that may be made by its manufacturer, is not guaranteed or endorsed by the publisher.
